# Durable Clinical Response to Pyrotinib After Resistance to Prior Anti-HER2 Therapy for HER2-Positive Advanced Gastric Cancer: A Case Report

**DOI:** 10.3389/fonc.2019.01453

**Published:** 2019-12-20

**Authors:** Le-Tian Huang, Jie-Tao Ma, Shu-Ling Zhang, Xiao-Han Li, Li Sun, Wei Jing, Jian-Zhu Zhao, Yan-Ru Wang, Cheng-Bo Han

**Affiliations:** ^1^Department of Oncology, Shengjing Hospital of China Medical University, Shenyang, China; ^2^Department of Pathology, Shengjing Hospital of China Medical University, Shenyang, China

**Keywords:** apatinib, gastric cancer, HER2, nivolumab, pyrotinib

## Abstract

**Background:** Patients with advanced gastric cancer, especially the HER2-positive type, have a poor prognosis; there is a paucity of effective anti-HER2 drug therapies in patients who develop resistance to trastuzumab.

**Case presentation:** We report the case of a 36-year-old male with HER2-positive gastric cancer with lung and liver metastases. The patient responded after treatment with trastuzumab combined with chemotherapy and attained a progression-free survival (PFS) of 17 months. Subsequently, the patient received apatinib that selectively inhibits the VEGFR2 and obtained an evident tumor response and a PFS of 8 months. When the disease progressed again, the regimen containing lapatinib failed. Then, the patient received treatment with nivolumab. However, he presented with hyper-progressive disease (HPD). Finally, he received a combination of capecitabine and pyrotinib, an irreversible dual TKI, acting on HER2 and EGFR. The tumor shrank markedly with this combination therapy. The mechanism of both HPD due to immunotherapy and the resistance to trastuzumab and lapatinib were investigated in this case. Loss of phosphatase and tensin homolog (PTEN) and new mutations of BRCA1 and KRAS were detected after resistance to trastuzumab and lapatinib.

**Conclusions:** For patients with HER2-positive advanced gastric cancer who have developed resistance to trastuzumab, pyrotinib is a promising new agent, which can be used as salvage therapy.

## Introduction

In recent years, use of targeted therapy against human epidermal growth factor receptor-2 (HER2) and immunotherapy against programmed cell death protein-1 (PD-1) have resulted in progress in the treatment of advanced gastric cancer. Moreover, small molecule tyrosine kinase inhibitors (TKI) (e.g., regorafenib and apatinib) targeting vascular endothelial growth factor receptor (VEGFR) have been investigated for their role in gastric cancer. A phase III randomized controlled trial (RCT) has shown that apatinib as third-line treatment for advanced gastric cancer significantly improves the objective response rate (ORR) and overall survival (OS), as compared with placebo (1). Based on this, apatinib was approved by the China Food and Drug Administration (CFDA) as third-line treatment for advanced gastric cancer.

The proportion of HER2-positive gastric cancers varies widely from 7 to 34% (2, 3). Trastuzumab is effective in improving the survival of patients with HER2-positive gastric cancer (4). However, there is no standard of care for trastuzumab-resistant advanced gastric cancer (5). Pyrotinib is an irreversible TKI targeting both HER2 and epidermal growth factor receptor (EGFR). In a phase II RCT, compared with lapatinib plus capecitabine, pyrotinib plus capecitabine significantly improved ORR (78.5 vs. 57.1%) and prolonged progression-free survival (PFS) (18.1 vs. 7.0 months) in patients with HER2-positive advanced breast cancer. Moreover, patients were able to benefit from pyrotinib, regardless of whether trastuzumab was administered previously (6). Based on this trial, CFDA approved pyrotinib for the treatment of HER2-positive advanced breast cancer (7). Currently, it is unclear whether pyrotinib is effective in trastuzumab-resistant gastric cancer. Herein, we report a case with HER2-positive advanced gastric cancer, who had previously been treated with trastuzumab, benefiting from apatinib and pyrotinib.

## Case Presentation

**Figure 1:**
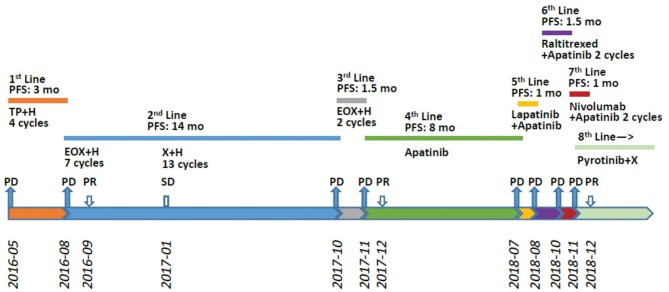
Treatment procedure after post-operative relapse. PFS, progression-free survival; H, trastuzumab; DP, docetaxel/cisplatin; X, xeloda (capecitabine); EOX, epirubicin/oxaliplatin/xeloda; PR, partial response; PD, progressive disease; mo, months.

**Figure 2:**
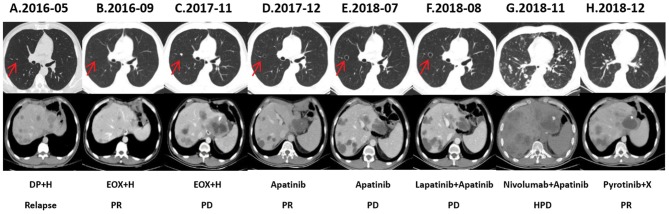
Chest and abdominal computed tomography (CT) scans. **(A)** CT (2016-05) demonstrates multiple liver and lung metastases before first-line chemotherapy (DP: docetaxel and cisplatin) combined with trastuzumab **(H)**. **(B)** Liver and lung metastases are reduced in size after second-line chemotherapy (EOX: epirubicin, oxaliplatin, and capecitabine) combined with trastuzumab. **(C)** CT (2017-11) demonstrates progressive disease (PD) in the lung and liver metastases when chemotherapy combined with trastuzumab failed. **(D)** CT (2017-12) reveals that a cavity is formed inside the pulmonary metastatic lesions, and the size of the liver metastases are reduced than before, after using fourth-line therapy (apatinib) for 1 month. **(E)** Lung and liver metastases are stable during apatinib treatment until 2018-07, when all the lesions progressed significantly. **(F)** After 4 weeks of oral lapatinib and apatinib treatment as fifth-line therapy, CT (2018-08) scans suggest new intrapulmonary nodules and intrahepatic metastases. **(G)** CT scans reveal hyper-progressive disease (HPD) and that the lesions are increased and enlarged significantly after treatment with nivolumab plus apatinib for 4 weeks. **(H)** CT scans (2018-12) show partial response (PR) in the hepatic and pulmonary metastatic lesions after using pyrotinib plus capecitabine for 1 month.

**Figure 3:**
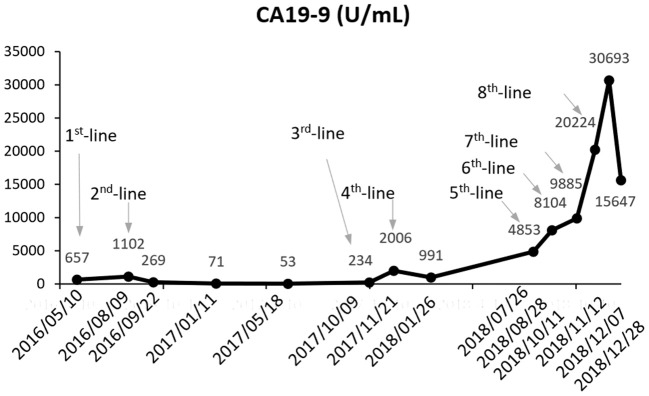
Change in cancer antigen 19-9 (CA19-9) (U/ml) levels during entire treatment.

A 36-year-old male patient was clinically diagnosed with gastric cancer and underwent D2 radical gastrectomy in October 2015. The histopathological diagnosis was moderately to highly differentiated adenocarcinoma (intestinal type) with HER2 amplification (FISH); TNM staging (7th edition) was pT4aN2M0 (stage IIIB). Subsequently, the patient underwent six cycles of adjuvant chemotherapy (oxaliplatin and capecitabine) without trastuzumab. Figures 1–3 illustrate the entire treatment procedure and corresponding changes in the lesions on computed tomography (CT) scan and changes in CA19-9 levels.

### First- and Second-Line Chemotherapy Combined With Trastuzumab

In May 2016, a CT scan demonstrated multiple liver and lung metastases; he was then initially treated with docetaxel, cisplatin, and trastuzumab. After four cycles, a CT scan in August 2016 showed that the intrapulmonary nodules were stable; the liver lesions, however, had enlarged markedly. He received second-line chemotherapy with EOX (epirubicin, oxaliplatin, capecitabine) combined with trastuzumab. Chest and abdominal CT examinations performed after two cycles showed partial remission in both the lung and liver lesions. After seven cycles, the regimen was switched to trastuzumab plus capecitabine, as maintenance therapy, until April 2017, when an abdominal CT showed slowly progressive lesions in the liver. Subsequently, he underwent transarterial chemoembolization twice and simultaneously continued with trastuzumab plus capecitabine for two more cycles, until October 2017, when CT scans demonstrated progressive disease (PD) in both the lung and liver metastases. From October 2017, the patient was rechallenged with two cycles of trastuzumab plus EOX, but this regimen failed to demonstrate any efficacy.

### Apatinib Monotherapy

From November 2017, the patient was treated with 750 mg of oral apatinib, once daily, as fourth-line therapy. One month later, CT showed that the intrahepatic lesions had markedly shrunken in size, and the intrapulmonary lesions showed either reduced density or cavity formation. During treatment with apatinib, the patient developed grade 2 adverse effects of leukopenia, hematuria, and anorexia, which subsided after symptomatic treatment.

### Lapatinib Combined With Apatinib

On July 26, 2018, a follow-up CT scan revealed that the lung and liver metastatic lesions had progressed significantly. He presented with the symptoms of cough, fatigue, and anorexia. The ECOG (Eastern Cooperative Oncology Group) score decreased to 2, and the quality of life (QOL) score was 39. Re-biopsy of the progressive lesions was suggested, but the patient refused. A regimen of lapatinib (1.25 g, once daily) combined with continuous apatinib (500 mg, once daily) was initiated. After 4 weeks of oral lapatinib and apatinib treatment, CT scans revealed new mediastinal lymph node metastasis, intrapulmonary nodules, and intrahepatic metastasis. Cough, fatigue, and anorexia persisted. In September 2018, the patient received raltitrexed combined with apatinib. However, the treatment failed after two cycles.

### Nivolumab Combined With Continuous Apatinib

In October 2018, a re-biopsy to the liver metastasis revealed a persistent histopathological diagnosis of adenocarcinoma. Subsequently, next generation sequencing (NGS) (543 genes; GeneCast Biotechnology Co. Ltd., Beijing, China) was performed. The detailed results before and after treatment with trastuzumab and lapatinib are listed in Table 1. Compared with the results before treatment with trastuzumab, NGS revealed a decreased gene copy number (GCN) of phosphatase and tensin homolog (PTEN), new mutations of BRCA1 and KRAS, and greater HER2 GCN. NGS also showed microsatellite stability (MSS) and moderate tumor mutational burden (TMB) (11.44/Megabase [Mb]). Immunohistochemistry (IHC) using both Ventana SP142 antibody and Dako 22c3 antibody was negative for PD-L1 expression. A regimen of nivolumab combined with continuous apatinib was given in October 2018. After 4 weeks, CT scans revealed that the tumors in the liver and lungs had increased and enlarged significantly. We calculated the tumor growth rate (TGR), before and during treatment, per month, and its variation (ΔTGR). The ΔTGR exceeded 50%, suggesting a hyper-progressive disease (HPD) and a poor prognosis (8).

**Table 1:** Results detected before treatment with trastuzumab and after resistance to treatment with trastuzumab and lapatinib.

**Genes**	**Before trastuzumab**			**After resistance to trastuzumab and lapatinib**			**Methodology**
	**Variations**	**Abundance**	**GCN**	**Variations**	**Abundance**	**GCN**	
HER2	Increased GCN		61.07	Increased GCN		100.9	NGS
BRCA1	Negative			p.Q1281Mfs^*^4	3.1%		NGS
PTEN	Negative			Decreased GCN		1.4	NGS
KRAS	Negative			p.G12C	21.7%		NGS
KRAS	Increased GCN		4.29	Increased GCN		3.7	NGS
PD-L1^*^	Negative	<1%^*^		Negative	<1%^*^		IHC
TMB	Moderate	5.08/Mb		Moderate	11.4/Mb		NGS
MSI	MSS/MSI-L			MSS/MSI-L			NGS
TP53	p.G244C	37.29%		p.G244C	51.1%		NGS
EGFR	Negative			Negative			NGS
STK11	Negative			Negative			NGS
MDM2	Negative			Negative			NGS

* *PD-L1 protein expression <1% for both tumor cells and tumor infiltrating immune cells*.

*HER2, human epidermal growth factor receptor-2; BRCA1, breast cancer susceptibility gene 1; PTEN, phosphatase and tensin homolog; KRAS, kirsten rat sarcoma viral oncogene; PD-L1, programmed cell death-ligand 1; TMB, tumor mutational burden; MSI, microsatellite instability; MSS, microsatellite stability; MSI-L, microsatellite instability low; TP53, tumor protein p53; EGFR, epidermal growth factor receptor; STK11, serine/threonine kinase 11; MDM2, mouse double minute 2 homolog; GCN, gene copy number; NGS, next generation sequencing; IHC, immunohistochemistry*.

### Pyrotinib Combined With Capecitabine

In November 2018, the patient was administered pyrotinib and capecitabine (pyrotinib 400 mg, once daily; capecitabine 1,000 mg/m^2^, bid, days 1–14, every 3 weeks). On the fifth day of treatment, the patient's dyspnea and cough gradually improved. After 2 weeks of treatment, the patient's dyspnea, dry cough, and fatigue completely subsided. After two cycles, chest and abdominal CT scans showed partial response (PR) in the pulmonary, mediastinal lymph node, hepatic, and peritoneal metastases. The patient tolerated treatment with pyrotinib well. Grade 1 diarrhea with no additional adverse events was observed and disappeared soon after symptomatic treatment. Until the submission of the case draft, the patient has survived for over 35 months since post-operative recurrence and now continues to receive the combination treatment of pyrotinib and capecitabine and the PFS is over 8 months.

## Discussion

Studies have shown that HER2 protein plays a critical role in the tumorigenesis, progression, and metastasis of HER2-positive gastric cancer, and portends a poor prognosis (9–11). Trastuzumab is presently the only drug with definite clinical evidence and proven efficacy in treating HER2-positive gastric cancer. The ToGA trial confirmed that chemotherapy combined with trastuzumab significantly improved ORR and OS for advanced HER2-positive gastric cancer compared with chemotherapy alone (4). The case we present responded to first-line trastuzumab treatment and attained a PFS of 8 months. After progression, the patient received multi-line chemotherapy successively without achieving remission, until the anti-angiogenic drug apatinib achieved a PR and a PFS of 8 months. Although subsequent treatment with lapatinib and immunotherapy failed, pyrotinib achieved apparent clinical remission, suggesting its ability to overcome prior resistance to anti-HER2 therapy. This underscores the fact that the HER2 signaling pathway has always been crucial for driving tumor progression.

Other anti-HER2 agents (e.g., lapatinib, afatinib, neratinib, dacomitinib, pertuzumab, and ado-trastuzumab emtansine) have not been validated for the treatment of HER2-positive gastric cancer with progression on or after trastuzumab-based therapy. In this case, we attempted to explore the mechanisms of resistance to lapatinib and/or trastuzumab by NGS. We speculate that acquired PTEN deletion (decreased GCN) is perhaps the main cause of drug resistance. It has been reported that low levels of PTEN may contribute to resistance of breast cancer cells to trastuzumab both *in vitro* and *in vivo*. Activated HER2 and downstream PI3K-Akt signaling pathway can be negatively regulated by PTEN molecule. Constitutively activated Akt via PTEN deletion leads to trastuzumab resistance (12–14). A retrospective study that included 129 patients with HER2-positive gastric cancer treated with first-line trastuzumab-based regimens, indicates that patients with loss of PTEN expression have a significantly shortened median PFS (4.5 vs. 12.4 months) and OS (12.3 vs. 28.9 months) compared with those with intact PTEN expression (15). In our case, pyrotinib has been effective, although the patient demonstrated PTEN deletion and resistance to trastuzumab and lapatinib. The reason for this is worth exploring.

Both pyrotinib and lapatinib are small-molecule TKIs that inhibit EGFR and HER2; however, lapatinib is a reversible TKI, whereas pyrotinib is irreversible. Primary resistance to lapatinib may be due in part to its reversible drug activity and incomplete inhibition of downstream pathways. Pyrotinib directly acts on the tyrosine kinase domain of the HER2 pathway and completely blocks downstream pathways activated by homodimers or heterodimers of EGFR, HER2, and HER4 on the tumor cell membrane. Monoclonal antibodies such as trastuzumab and pertuzumab only act on the HER2 pathway (16). Preclinical studies that investigated the inhibitory activity of various anti-HER2 drugs in HER2-positive gastric cancer cell lines (NCI-N87) found the IC_50_ of lapatinib, neratinib, and pyrotinib to be 10, 0.6, and 1.0 nmol/L, respectively (17, 18), suggesting that the antitumor activity of lapatinib in HER2-positive gastric cancer is evidently weaker than that of irreversible TKI pyrotinib. A phase I study of pyrotinib in the treatment of HER2-positive gastric cancer (NCT03480256) is currently underway; however, no data have been reported as yet.

Of note, as the fourth-line therapy in this case, apatinib achieved PR and a PFS of 8 months. There is no clinical trial reporting the efficacy of apatinib in specific HER2-positive gastric cancer. Our case suggests that patients with HER2-positive gastric cancer can still benefit from apatinib after resistance to trastuzumab. Further clinical trials will be needed to verify and validate the efficacy of apatinib in advanced HER2-positive gastric cancer.

The results of NGS and IHC before treatment with the immune checkpoint inhibitor (ICI), nivolumab, suggest that some molecular biomarkers (e.g., PD-L1, TMB, MSI, TP53, KRAS, BRCA1, STK11, MDM2) may be related to the efficacy of ICI (Table 1). Retrospective data from clinical studies have shown that patients with lung adenocarcinoma harboring both TP53 and KRAS mutations have a significant survival benefit when treated with ICI (19, 20). Unfortunately, the patient developed HPD after two cycles of nivolumab. A randomized, placebo-controlled, phase III trial shows nivolumab can prolong median OS by 1 month (5.26 vs. 4.14 months) in PD-L1-positive (≥1%) advanced gastric cancer (21). However, patients with HER2-positive gastric cancer were excluded from enrolment in this trial; hence, it is unclear whether these HER2-positive patients were prone to developing HPD. A clinical study exploring the predictors of HPD after the use of nivolumab in advanced gastric cancer found that the frequencies of HPD in HER2-positive and negative gastric cancers are 23.4% (11/47) and 14.3% (2/14) (*p* = 0.71), respectively (22). This suggests that HER2-positive status may not be associated with HPD. Moreover, previous studies have shown that loss of PTEN promotes resistance to T cell–mediated immunotherapy (23, 24). In view of this, ICI may not be a good option for patients who are resistant to HER2 agents with loss of PTEN. Instead, pyrotinib could be proposed even in patients with loss of PTEN. For patients who are resistant to pyrotinib, agents acting on the PI3k/PTEN-Akt-mTOR pathway (e.g., everolimus), could be tried as follow-up combination treatment.

## Conclusion

In our case, pyrotinib was administered to treat advanced HER2-positive gastric cancer after resistance to prior anti-HER2 therapy and the patient has achieved an evident treatment response. Pyrotinib is a promising novel agent, which can be used as a salvage therapeutic drug for anti-HER2-resistant gastric cancer. Further studies are necessary to validate this result.

## Ethics Statement

Written informed consent was obtained from the patient for publication of this case report and any potentially identifying information and images.

## Author Contributions

All authors have read and approved the final manuscript. C-BH designed the report. L-TH wrote the manuscript. C-BH and J-TM were responsible for the conception and revision of the manuscript. X-HL undertook the pathological diagnosis. C-BH, J-TM, L-TH, S-LZ, and LS carried out the clinical management of the patient. L-TH, S-LZ, and LS collected the patient's clinical data. WJ, J-ZZ, and Y-RW analyzed the data.

### Conflict of Interest

The authors declare that the research was conducted in the absence of any commercial or financial relationships that could be construed as a potential conflict of interest.
